# Comparison of anatomically informed class solution template trajectories with patient‐specific trajectories for stereotactic radiosurgery and radiotherapy

**DOI:** 10.1002/acm2.13765

**Published:** 2022-09-02

**Authors:** John David Lincoln, Robert Lee MacDonald, Brian Little, Alasdair Syme, Christopher Grant Thomas

**Affiliations:** ^1^ Department of Physics and Atmospheric Science Dalhousie University Halifax Nova Scotia Canada; ^2^ Department of Medical Physics Nova Scotia Health Authority Halifax Nova Scotia Canada; ^3^ Department of Radiation Oncology Dalhousie University Halifax Nova Scotia Canada; ^4^ Beatrice Hunter Cancer Research Institute Halifax Nova Scotia Canada; ^5^ Department of Radiology Dalhousie University Halifax Nova Scotia Canada

**Keywords:** 4pi, automation, noncoplanar, optimization, SRS/SRT, trajectory optimization

## Abstract

Class solution template trajectories are used clinically for efficiency, safety, and reproducibility. The aim was to develop class solutions for single cranial metastases radiotherapy/radiosurgery based on intracranial target positioning and compare to patient‐specific trajectories in the context of 4π optimization. Template trajectories were constructed based on the open‐source Montreal Neurological Institute (MNI) average brain. The MNI brain was populated with evenly spaced spherical target volumes (2 cm diameter, *N* = 243) and organs‐at‐risk (OARs) were identified. Template trajectories were generated for six anatomical regions (frontal, medial, and posterior, each with laterality dependence) based on previously published 4π optimization methods. Volumetric modulated arc therapy (VMAT) treatment plans generated using anatomically informed template 4π trajectories and patientspecific 4π trajectories were compared against VMAT plans from a standard four‐arc template. Four‐arc optimization techniques were compared to the standard VMAT template by placing three spherical targets in each of six anatomical regions of a test patient. This yielded 54 plans to compare various plan quality metrics. Increasing plan technique complexity, the total number of OAR maximum dose reductions compared to the standard arc template for the 6 anatomical classes was 4+/−2 (OFIXEDc) and 7+/−2 (OFIXEDi). In 65.6% of all cases, optimized fixed‐couch positions outperformed the standard‐arc template. Of the three comparisons, the most complex (OFIXEDi) showed the greatest statistical significance compared to the least complex (VMATi) across 12 plan quality metrics of maximum dose to each OAR, V12Gy, total plan Monitor Units, conformity index, and gradient index (*p* < 0.00417). In approximately 70% of all cases, 4π optimization methods outperformed the standard‐arc template in terms of maximum dose reduction to OAR, by exclusively changing the arc geometry. We conclude that a tradeoff exists between complexity of a class solution methodology compared to patient‐specific methods for arc selection, in the context of plan quality improvement.

## 1 INTRODUCTION

Recent advances in radiotherapy have enabled the ability to automate treatment planning procedures while ensuring there are no losses in dosimetric plan quality compared to conventionally planned treatments.^1^, ^2^, ^3^, ^4^ In cranial stereotactic radiosurgery/radiotherapy (SRS/SRT), an example of one such automation is the HyperArc product by Varian Medical Systems (Palo Alto, CA, USA).^5^ HyperArc offers a push button solution to cranial SRS/SRT by employing a noncoplanar template solution of arcs, published by Clark et al.^6^ This arc‐geometry template will be defined for use in this research as “the standard arc template” for sake of comparison at our institution. The standard arc template includes four arc trajectories: a full coplanar arc, a partial vertex arc, and two partial noncoplanar arcs where the treatment couch is offset by 45° to each side of the 0° couch position. While this arc geometry can be used as a starting point for cranial SRS/SRT volumetric modulated arc therapy (VMAT), it does not consider the cranial anatomy from a given beams‐eye‐view (BEV). BEV‐based beam selection, whether it be fixed‐port intensity modulated radiotherapy or VMAT, has been shown to be an effective tool for reducing normal tissue doses^7^, ^8^, ^9^, ^10^, ^11^, ^12^ because it assists in the selection of beams that do not result in BEV overlap between targets and organs‐at‐risk (OARs), thus avoiding irradiation of those OARs.

A step between the extremes of patient‐specific optimization and a fixed‐arc template geometry solution like HyperArc could be a class solution based on patient anatomy. Class solutions can facilitate radiotherapy treatment planning by giving a template to planners upon which the treatment plan can be further optimized. Templates have the added benefit of reducing variation in the planning process, which can lead to improved efficiency, and reproducibility due to their familiarity to the planner. The HyperArc solution discussed above is an example of such a template, where the planner is removed from the arc selection decision but can also modify it as desired. Class solutions have been used in prostate treatment planning,^13^, ^14^ for whole brain irradiation,^14^ and for SBRT of spinal indications.^15^ Podgorsak et al. and Wilson et al. proposed the utility of noncoplanar class solutions for SRS^16^, ^17^; however none of these preceding solutions have used an anatomical approach such as an overlap‐guided methodology for geometry selection. In an era where treatment plan automation is becoming more prevalent, increasingly sophisticated methods are needed to ensure that OARs are spared as much as reasonably achievable. Moreover, these plans also need to be robust in different geometries. Given that there is a precedent for planners using templates in SRS and SBRT, a natural step forward is to include anatomical information to create such a template. These templates could then be compared with the template of Clark et al. and patient‐specific trajectories. Each of these planning techniques can be considered in terms of a tradeoff between their degree of complexity in geometric arc selection and ability to improve plan quality.

In this study, a novel method was developed to create anatomically informed template trajectories (class solutions) for cranial SRS/SRT by employing 4π optimization methods based on the methods of MacDonald et al.^8^ to calculate BEV overlap maps for each region of a segmented cranial template. To allow these plans to be optimized in the clinical treatment planning system (TPS), navigation methods were restricted to fixed couch (nonsimultaneous motion of couch and gantry) optimizations that would allow for VMAT optimization in a clinical TPS once optimized arc geometries were determined. This methodology also facilitates ease of clinical translation with minimal change to current practice.

The objectives of our study were as follows:

1. Develop a novel methodology to create anatomically informed template trajectories.
2. Compare an anatomically informed class solution against a patient‐specific map navigation.
3. Compare methods to the standard arc template used in SRS/SRT.

The method chosen for fixed‐couch navigation optimization is derived from previously published work of MacDonald et al.^18^ that chooses the best of up to four fixed‐couch arcs unconstrained by gantry arc span.

This work serves to investigate whether 4π optimized class solutions would be sufficient when treating single metastases in the brain with SRS/SRT, using increasingly complex methods of arc selection to reduce doses to OAR compared to a four‐arc geometric template. Sampling 4π space with an anatomically informed class solution approach will give a better sense of anatomical relevance compared to a standardized geometric arc placement. The class solutions should be anatomically informed to account for relevant anatomies and differential overlap in separate regions of the brain. Anatomically informed class solution templates will also be compared to patient‐specific arc trajectories to investigate the tradeoff between customization and efficiency in arc selection. Finally, the methodology herein is generalizable to any anatomical location in the body.

## 2 METHODOLOGY

### 2.1 Creating an accurate anatomical model for cranial SRS/SRT template trajectories

All work done to create an accurate anatomical model and associated trajectories was performed in MATLAB version 2018b (MathWorks, Natick, MA). To develop template trajectories that were anatomically informed for cranial cases, the Montreal Neurological Institute's (MNI) average brain was used^19^ as a generic brain. This anatomy is based on automated co‐registration of 305 T1‐weighted MRI scans and aligned in Talairach space.^19^ Relevant OARs were contoured and merged into a singular avoidance structure, while the normal brain tissue was taken as the outer contour of the MNI brain with the targets subtracted. A singular avoidance structure combining all OAR was chosen to solely focus on amount of BEV overlap with a given target, removing bias between potential region of interest (ROI) combinations. OARs incorporated into this model were brainstem, chiasm, left and right optic nerves, left and right eyes, and left and right lenses. This methodology was a modified version of the BEV overlap calculation performed by MacDonald et al.^8^ where fractional overlap is dictated by each OAR individually. Collision zones on the maps were all manually measured as per MacDonald et al..^8^

**FIGURE 1:**
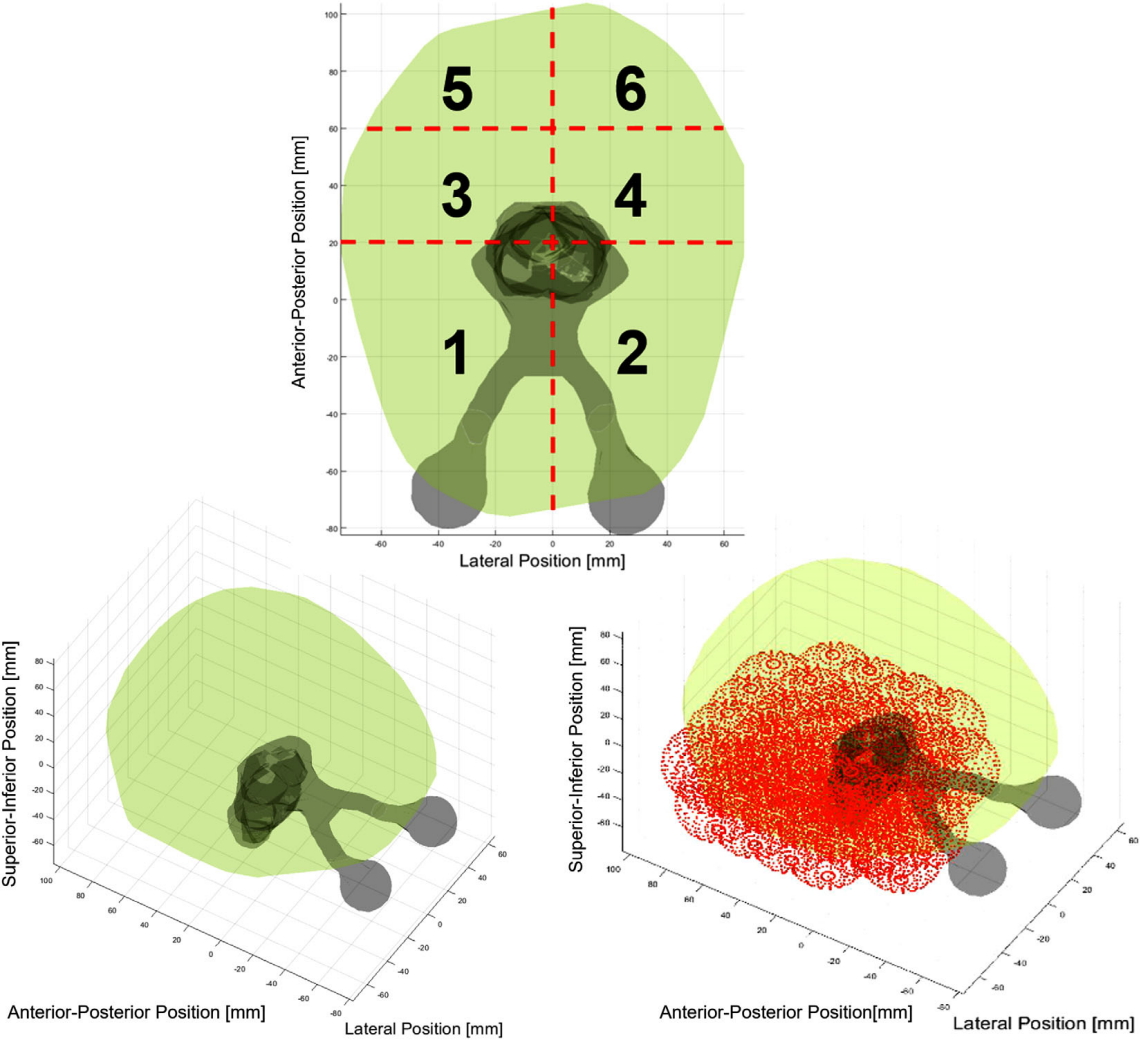
(Left) The Montreal Neurological Institute (MNI) brain^15^ outer contour (light green), with summed organs‐at‐risk (OARs) structures (grey). (Right) Mid‐way through the systematic placement of equally spaced, equally sized 2‐cm diameter spherical targets, constrained to be inside the normal brain outer contour. (Bottom) Segmentations for the MATLAB simulations: frontal, medial, and posterior segmentations are shown, each with lateral dependence.

The next step in creating the model was to populate the MNI brain with targets. For each target that was placed inside the brain, an overlap map was calculated based on the method of MacDonald et al.^8^ for all possible couch and gantry angle combinations at 1‐degree resolution. The MNI brain was filled by systematically placing 243 equally sized (2 cm diameter), equally spaced (2 cm apart) targets inside the outer contour (normal brain tissue) (Figure 1).

The brain was then segmented into six distinct anatomical segments to enable anatomically informed template trajectories to be calculated for each. These segments were frontal, medial, and posterior, each with lateral dependence. The MNI brain was segmented according to the following rules, measured in accordance with Figure 1:

1. Divide left and right hemispheres along the lateral position (*x* = 0 mm). Right hemisphere was denoted as negative lateral position values, while left hemisphere was denoted as positive lateral position values.
2. Frontal segments spanned from the most negative (∼−80 mm) anterior–posterior position of the outer contour to the anterior‐posterior position of 20 mm.
3. Medial segments spanned from the 20 mm anterior–posterior to 60 mm anterior–posterior.
4. Posterior segments spanned from 60 mm anterior–posterior to the most positive (∼100 mm) anterior–posterior position.
5. Given lateral and anterior–posterior limitations that define a two‐dimensional projection, include all superior‐inferior values encompassed by these limitations to define three‐dimensional segment.

### 2.2 Segmenting the anatomical model to calculate overlap maps

Of the 243 synthetic targets generated during the simulation, an average overlap map was needed for each of the six anatomical segments, which could then be navigated. These were determined by calculating the maximum amount of overlap for every couch and gantry angle combination on a per‐anatomical segment basis. This ensured a conservative overestimate of all overlap scores for each anatomical segment's template map.

With these anatomically informed template maps, anatomically informed class solution template trajectories were calculated using methods based on previously published 4π methodologies. The method was proposed by MacDonald et al.^18^ that uses optimal pathfinding to choose the best fixed‐couch arcs given any overlap map. Constrained solely by the Eclipse (Varian Medical Systems, Inc., Palo Alto, CA) TPS version 13.6 restrictions that dictate no VMAT arcs can span less than 30 degrees,^20^ this optimal fixed‐couch (OFIXED) algorithm will automatically choose sub arcs that are not limited to being of equal length with fixed‐couch positions that offer the lowest overlap for a given overlap map. In this research, OFIXED was further constrained to match the sub arc limit of four, in agreement with the total number of couch positions used in the standard arc template.

The fixed‐couch positions and arc geometries were used in the generation of VMAT plans using Eclipse version 13.6.

### 2.3 Generating synthetic cases in Eclipse for planning

Once the anatomically informed template trajectories were calculated, the Eclipse contouring tool was used to create synthetic spherical targets inside a previously treated patient's cranial dataset, different than the MNI brain, which was segmented according to the same method used in the simulations of Section 2.1. Three spherical targets (diameters 0.5 cm, 1 cm, and 2 cm) were placed in each segment, one at a time, yielding a total of 18 synthetic cases.

After all the synthetic cases were created, the structure set was exported from Eclipse so patient‐specific overlap maps could be calculated in Matlab. Once each of the 18 patient‐specific overlap maps was calculated, OFIXED algorithm was used to generate fixed‐couch arc trajectories for them.

### 2.4 Eclipse planning procedure

To establish whether dosimetry is impacted by the type of 4π fixed couch sub arc optimization, comparisions were performed between plans that were VMAT optimized after performing fixed‐couch optimizations. These optimizations were based on overlap from anatomically informed class solution overlap maps, and patient‐specific overlap maps.

For all 18 synthetic cases, three for each of the six classes, three plans were created with VMAT optimization, yielding a total of 54 plans. Overall, these three different planning methodologies for comparison were identified in order of increasing complexity as:

1. VMATi = The standard arc template.
2. OFIXEDc = Anatomically informed class solution with fixed‐couch trajectory generated from the OFIXED algorithm.
3. OFIXEDi = Patient‐specific solution with fixed‐couch trajectory generated from the OFIXED algorithm.

Each plan had a prescription dose of 2400 cGy prescribed to the 90% isodose level, to be delivered in a single fraction to the synthetically created spherical planning target volume (PTV). The OARs considered for the VMAT optimization were left and right eyes, left and right lenses, brainstem planning‐risk‐volume (PRV), optic chiasm PRV, and left and right optic nerve PRVs. The PRV included in these optimizations were 2‐mm expansions of their respective OAR, and all OARs were subjected to upper dose VMAT optimization objectives. These optimization objectives instructed the VMAT optimizer to satisfy the objective that no percent of the total volume of an OAR should receive more than a specific dose value. Optimization objectives are inputs to the VMAT optimizer that attempt to drive the optimization into satisfying certain clinical constraints. In this manuscript, we use optimization objectives to first ensure adequate coverage of the PTV with the prescription dose, and then wherever possible to decrease maximum doses to OAR below their clinical tolerances.

To calculate upper dose optimization objectives for each OAR, distances were measured from the synthetic spherical PTV in each case to the OAR in every slice of the CT simulation dataset. Upper dose optimization objectives were taken as the prescription dose (2400 cGy) minus 10%/mm multiplied by the minimum distance of the OAR to the PTV, to systematically consider dose fall off when the PTV was proximal to OAR.^21^, ^22^ For example, if the brainstem was 1.7 mm proximal to the PTV, the upper dose optimization objective was set to:

(1) $$\begin{array}{ccc} 100\%Rx & - & 1.7\;\left[\mathrm{mm}\right]*10\;\left[\frac{\%Rx}{\mathrm{mm}}\right]=\;100\%Rx\\ & & -17\%Rx\;=\;83\%Rx \end{array}$$

Therefore, in this case, the brainstem PRV upper dose optimization objective would be such that 0% of the brainstem PRV volume could receive more than 83% of the prescribed 2400 cGy dose, 1992 cGy.

In the cases where the OAR was more than 10 mm from the PTV, the upper dose optimization objective was set to a value of 150 cGy, to minimize the dose to the OAR as much as possible without placing unreasonable demands on the VMAT optimizer. Here, we define a “proximity constrained OAR” as any OAR that was less than 10 mm from the PTV, and thus required an upper dose optimization objective to be calculated from Equation 1. A table detailing all proximity constrained OARs for each plan can be found in the Supporting Information (Supporting Information 8).

Furthermore, as a tool to try to maximize dose conformity to the target, a surrounding symmetrical tuning ring structure was created for each PTV with an outer diameter of 3 cm and an inner diameter of 1 cm. The tuning ring upper dose optimization objective was set at 0% volume to receive 33% of the 2400 cGy prescription dose (800 cGy).

Finally, when optimizing the PTV, upper and lower dose optimization objectives were applied to ensure that the entire PTV volume (>99%) received the prescription and to ensure that any hotspot was limited to no more than 15% of the prescription.

General VMAT optimization parameters included an automatic normal tissue optimization set to a priority of 175 and VMAT optimization grid set to “fine” (1.25 mm) within the VMAT progressive optimization algorithm version 13.623.^20^ When performing the final dose calculation algorithm (AAA version 13.623) for all plans after VMAT optimization,^23^ the dose calculation grid size was set to 1.5 mm, the closest option to the VMAT optimization grid.

Moreover, the objective of this research was to create a methodology that could be reproduced through automation. Each plan was optimized only once to ensure we would be comparing effects of only changing arc trajectories based on the method used for arc selection. This was further ensured by maintaining dose optimization objectives between plans and only changing arc geometries.

### 2.5 Dose comparison and plan quality comparison

Following VMAT optimization and dose calculation for all plans, the two categories chosen for comparison were OAR maximum doses and other plan quality metrics. Dosimetric comparison between plans was a comparison of maximum dose as the metric of interest for various OARs, as the brain is a structure comprised of many serial organs. Plan quality comparisons were performed using the volume of the normal brain, excluding the target, that receives 12 Gy (V12Gy); total plan monitor units (MUs); plan conformity index (CI)^24^; and plan gradient index (GI).^25^ The definition of CI used in this research was the Paddick conformity index shown in Equation 2,^24^ while the definition of GI used in this research was the Paddick dose gradient calculation shown in Equation 3.^25^

(2) $$\mathrm{CI}\;=\frac{V_{T,\mathrm{ref}}}{V_{T}}\;*\frac{V_{T,\mathrm{ref}}}{V_{\mathrm{ref}}}$$

In this definition, *V_T,_*_ref_ is the volume of the target receiving a dose equal to or greater than the reference dose, *V_T_* is the volume of the target, and *V*_ref_ is the volume receiving a dose equal to or greater than the reference dose.^24^ In this work, the reference dose was chosen to be the prescription dose of 2400 cGy.

(3) $$\mathrm{GI}\;=\frac{V_{\left(\frac{Rx}{2}\right)\frac{Rx}{2}}}{V_{Rx}}\;$$

In this definition, the gradient index is defined as the ratio of the volume of half the prescription isodose, to the volume of the prescription isodose.^25^

Maximum dose comparisons were performed between classes for all the plans corresponding to each anatomical segment. This consisted of averaging over the three targets placed in each segment. This allowed for six sets of maximum dose comparisons to be performed.

Plan quality metrics were compared across each of the three planning techniques by investigating the average difference in each metric. Each planning technique was performed on the 18 distinct cases described above. This results in a series of multiple independent comparisons of 12 separate plan quality metrics.

To account for this when performing statistical testing, a *p*‐value was calculated via a Wilcoxon‐Signed Rank Test for each metric inside a plan technique comparison, as the data were not normally distributed. Each test was two tailed. Comparisons were made for three planning techniques, thus three comparisons (three choose two) to perform for each of the 12 plan quality metrics, yielding 36 comparisons in the dataset. A full Bonferroni correction here would give a corrected significance level of 0.05/36 = 0.00139, which given the comparatively small sample size (*N* = 18) is extremely conservative. The Bonferroni corrected significance level was taken as 0.05/12 = 0.00417 (Bonferroni corrected significance per comparison). A full table of plan technique comparison as well as a full table of *p*‐values is given in the Supporting Information (Supporting Information 6 and 7).

## 3 RESULTS

### 3.1 Overlap maps generated from segmenting anatomical model

**FIGURE 2:**
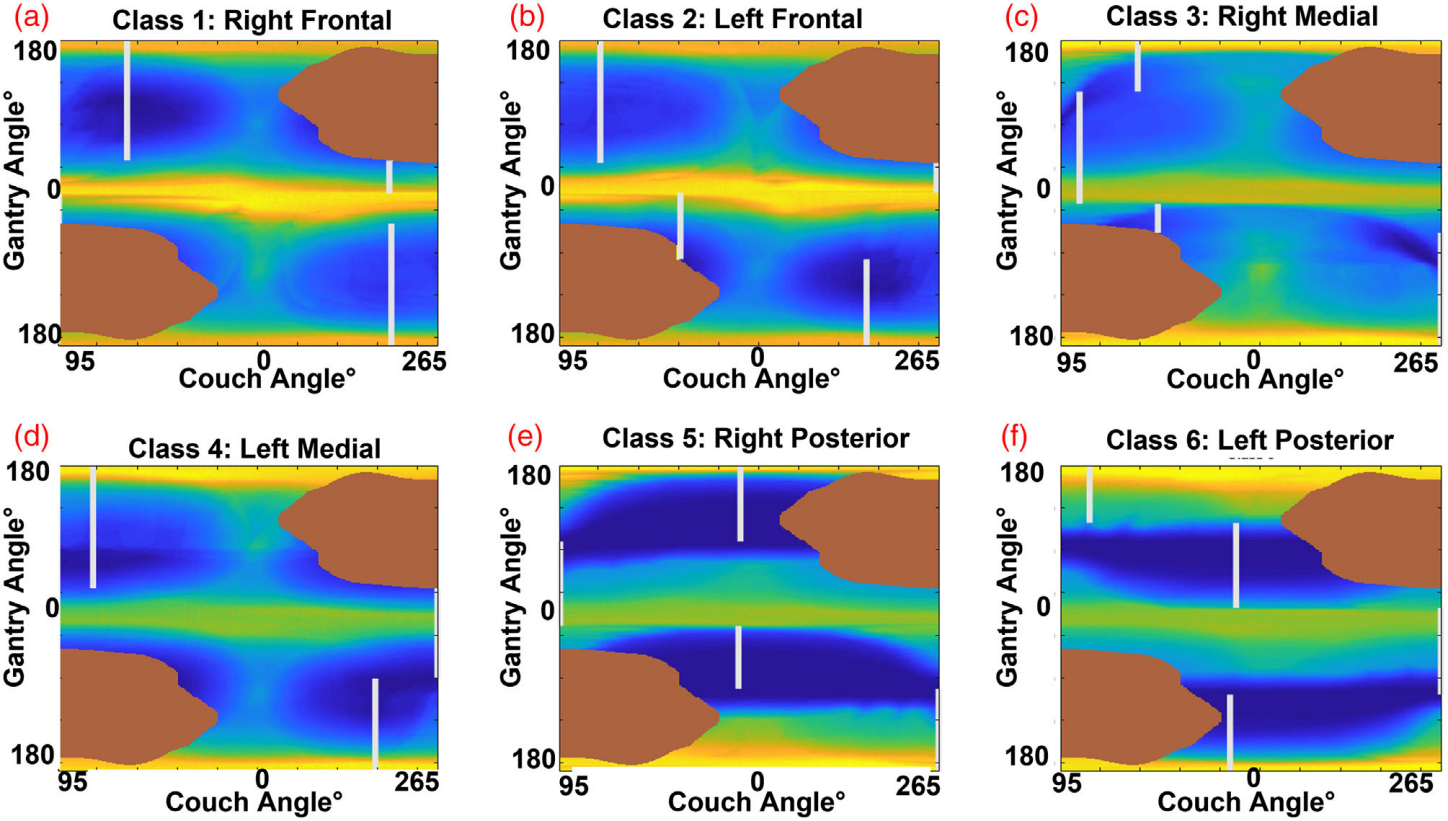
Trajectory class solutions for each of the ROI template maps denoted by “Class 1—6.” Brown regions were manually measured collision zones,^8^ white lines are fixed couch trajectories measured using OFIXED algorithm. Regions of high BEV overlap between organs‐at‐risk (OARs) and the target are yellow, while dark blue indicates regions of low overlap.

Performing calculations described in Section 2.2 yields the following anatomically informed class solutions and corresponding overlap maps (Figure 2).

### 3.2 Intraclass maximum dose comparison

**FIGURE 3:**
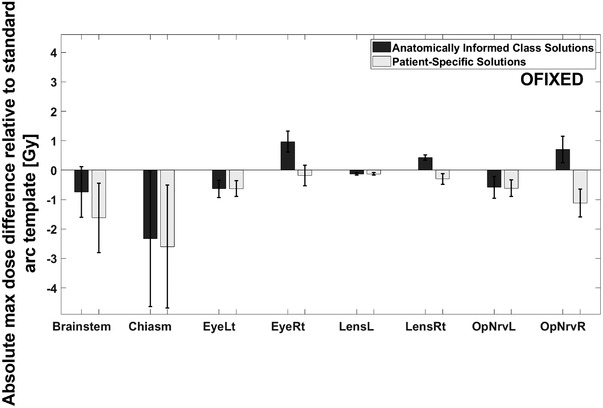
The results from anatomically informed class solution (Right Frontal segment). Dark bars indicate the results from the anatomically informed class solution trajectories, while the light bars indicate the results from patient‐specific trajectories. Dosimetric results are shown when applying trajectories using the OFIXED algorithm.

The following results in Figure 3 illustrate the average maximum dose difference relative to the standard arc template, over the three synthetic targets for an example anatomical class. In this case, anatomical “Class 1” was chosen, which corresponds to the “Right Frontal” overlap map of Figure 2a. All error bars are the standard error of the mean. A negative value in the bar plot indicates a reduction in average maximum dose as compared to the standard arc template. Results for all other anatomical classes can be found in the Supporting Material (Supporting Information 1–5).

In the plot of the results from the right frontal segment (Figure 3), for five and eight OARs, the OFIXED algorithm generated trajectories lowered maximum dose relative to the standard arc template using the class solution (OFIXEDc) and patient‐specific solution (OFIXEDi), respectively.

The results for all anatomical classes can be summarized in Table 1, where the number of OAR dose reductions can be realized based on what planning technique was used.

**TABLE 1:** Total number of organs‐at‐risk (OARs) (of the eight considered) that showed maximum dose reductions relative to the standard arc template for the planning techniques used in this work. Mean and standard deviation across classes were rounded to the nearest integer

	Class 1	Class 2	Class 3	Class 4	Class 5	Class 6	
**OFIXEDc**	5	3	5	5	2	2	**4 ± 2**
**OFIXEDi**	8	3	8	8	7	7	**7 ± 2**

The patient‐specific results were almost always at least as good if not better than the class solution, and in approximately 70% of cases, any optimized couch positions outperformed the standard arc template.

### 3.3 Max dose comparisons between arc selection techniques

To perform the multiple independent comparisons that arise from the trajectory selection techniques used in this research, a table was created that accounts for the three comparisons for the 18 specific cases. This table of planning metrics and associated significance values can be found in the Supporting Material (Supporting Information 6 and 7) but will be summarized based on the objectives of this research.

To compare fixed‐couch arc selection to the standard arc template, comparisons were performed with the standard arc template, detailed in Table 2.

**TABLE 2:** Number of organs‐at‐risk (OARs) maximum dose reductions when comparing every planning technique with the standard arc template (*N* = 18). Significance level Bonferroni corrected to *p* = 0.00417

Technique	Number of max dose reductions to OAR	Number of significant max dose reductions to OAR
OFIXEDc	4	0
OFIXEDi	8	3

When compared to the standard arc template averaging over the 18 independent cases, significant reductions are found for the patient‐specific optimization (OFIXEDi). These techniques also reduce maximum dose for each OAR, while class solutions (OFIXEDc) reduced maximum dose to four OARs (not statistically significant).

When comparing the class solution method to a patient‐specific method, eight OARs achieved maximum dose reductions for the OFIXED comparison but were not statistically significant.

### 3.4 Plan quality metrics comparisons between arc selection techniques

Like the max dose metrics shown in Section 3.3, the plan quality metrics of V12Gy, plan MUs, conformity index, and gradient index were compared for all planning techniques independently. In line with the comparison with the standard arc template (Table 2), Table 3 shows the summary of plan quality.

**TABLE 3:** Effect on plan quality when comparing every planning technique with the standard arc template (*N* = 18). Significance level Bonferroni corrected to *p* = 0.00417. Checkmark indicates meeting the criteria of improvement, X indicates worsening. * Indicates significance threshold met for either improvement or worsening

Technique	V12Gy reduced	Monitor units reduced	Conformity improved	Gradient improved
OFIXEDc	X	X	√	X*
OFIXEDi	X*	X	√	X

**FIGURE 4:**
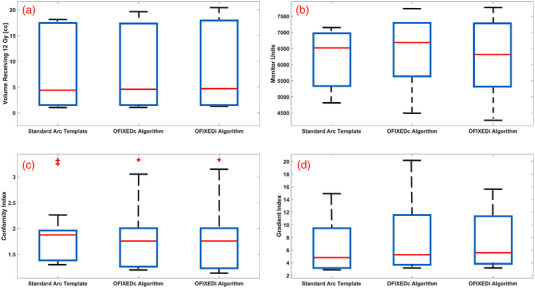
The absolute values for each planning technique (Standard Arc Template, OFIXEDc, and OFIXEDi) for V12Gy **(a)**, total plan monitor units **(b)**, conformity index **(c)**, and gradient index **(d)**. Median values are denoted by the solid red lines, while interquartile range is denoted by the surrounding blue box. Upper and lower quartiles are denoted by the dashed lines.

Figure 4 summarizes this comparison with the absolute values from each plan.

When compared to the standard arc template, conformity index improved in three of the four planning techniques, while all other plan quality metrics favoured the standard arc template, the majority of which were not statistically significant. A significant favouring for the standard arc template was found for V12Gy compared to OFIXEDi and for the gradient index compared to OFIXEDc.

When considering the comparison between class solution methods and patient‐specific methods, class solutions demonstrate superior conformity and V12Gy at the expense of inferior gradient indices. The majority of plan quality comparisons were not statistically significant.

### 3.5 Impact of OAR proximity on class solution effectiveness

**FIGURE 5:**
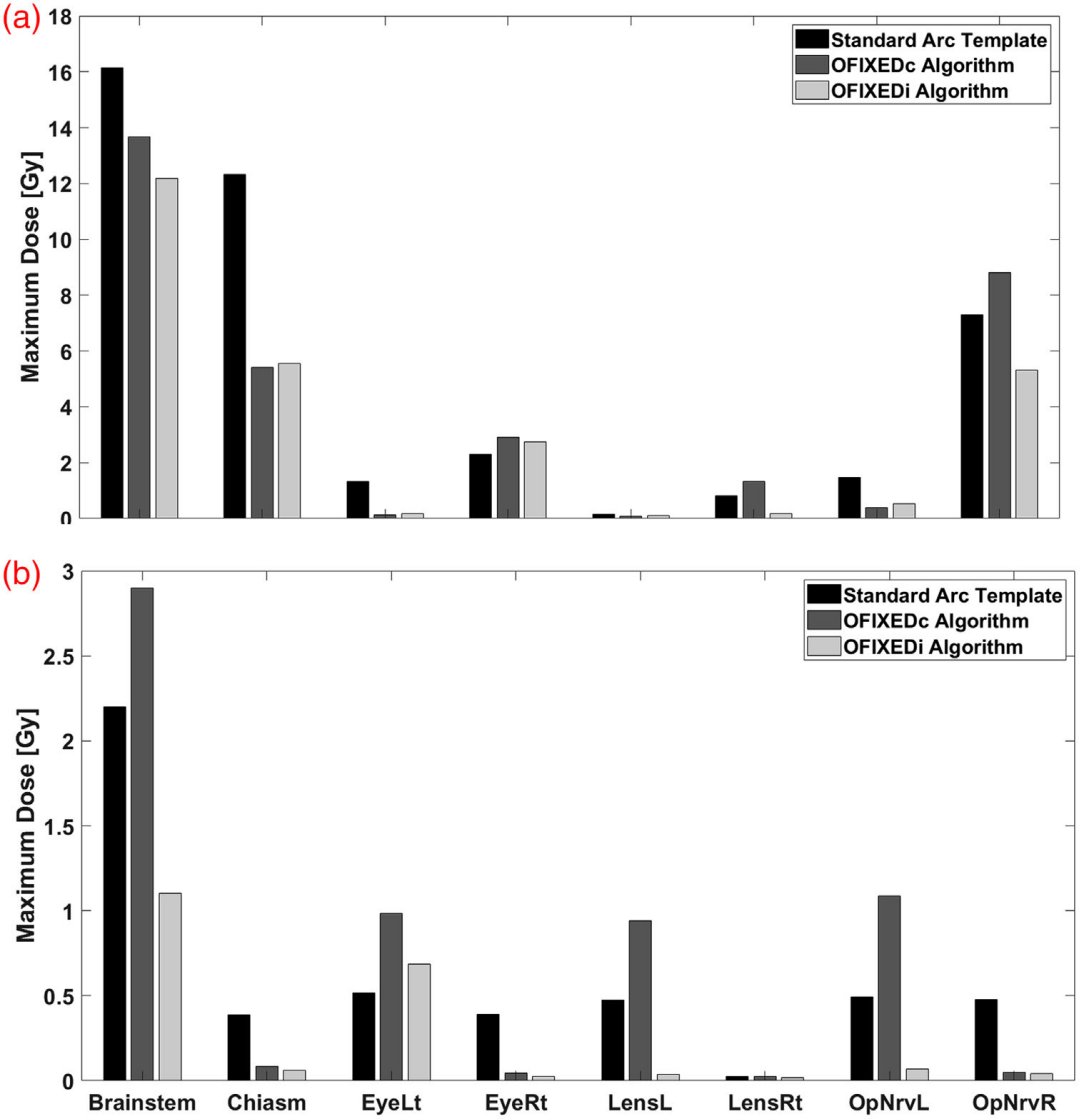
(a) The raw maximum dose results from a case placed in anatomical class 1 (2‐cm diameter spherical synthetic target, right frontal) proximal to brainstem, chiasm, and right optic nerve for all planning techniques. (b) A similar plot but from a case placed in anatomical class 6 (1‐cm diameter spherical synthetic target, left posterior) not proximal to any organs‐at‐risk (OARs)

An example comparison between two independent plans with considerably different proximity considerations on their respective OARs is shown in Figure 5, where a case with many proximal OARs was compared next to a case with no proximal OARs (class 1 versus class 6).

In Figure 5a, trajectory optimization relative to the standard arc template reduces maximum dose to OARs brainstem and chiasm, which were both considered as proximal OAR based on Equation 1, while only patient‐specific trajectory optimizations allow for the reduction of right optic nerve dose. This is contrasted with Figure 5b where various plan optimization techniques yield higher maximum doses to OARs than the standard arc template, which are not proximity constrained.

A table is included in the Supporting Material that summarizes all maximum dose differences and new significance values based on filtering cases that had proximity constraints (Supporting Information 9). Of the 18 independent cases, 11 required considerations of proximity of the PTV to one or multiple OAR. Nine of these were brainstem constraints, four were optic chiasm constraints, and two constraints each for right and left optic nerves, respectively. These yielded larger average maximum dose differences for all OAR where proximity was considered, respectively, but did not change significance.

## 4 DISCUSSION

The aim of this research was to perform a comparison of trajectories from noncoplanar (4π) optimization in the context of developing anatomically informed class solution template trajectories for cranial SRS/SRT. These trajectories were compared to our institutional standard arc template geometry. The purpose here was to demonstrate dosimetric and plan quality differences that arise from variations in arc selection, not planner quality. Therefore, a robust repeatable procedure was used to highlight these differences.

Fixed‐couch arc trajectories that conformed to the restrictions of the Eclipse TPS were calculated for the dosimetric comparisons. The method for arc selection has been previously compared to the standard four‐arc cranial template.^18^ Moreover, this comparison was performed using the framework of anatomically informed class solution template overlap maps for six cranial anatomical segments and patient‐specific overlap maps. These comparisons provide insight into whether an anatomically informed class solution template informed by optimized fixed‐couch arcs is sufficient for cranial SRS/SRT planning.

Synthetic cases simulating single metastases that occur in various parts of the brain were taken as a starting point for developing the anatomically informed class solutions. The aim of this research was not to examine multiple metastases cases, which would involve more complex considerations in the overlap calculations. Comparing arc selection techniques between different regions of interest was facilitated with the flexibility to randomly place a synthetic target inside a known anatomical segmentation, thus allowing for consistent statistics (six classes each with three synthetic spherical targets).

First, when considering Figure 1, although the MNI average brain was not completely symmetric, it seemed reasonable to expect mirrored behavior from the lateral dependence of anatomical segments. For example, the overlap maps for classes 1, 3, and 5 (right hand side) should be approximately the flipped versions of classes 2, 4, and 6, respectively. However, the optimized arc trajectories were not simply the flipped versions between classes, implying that there could be differences when performing dosimetric comparisons, and thus a limitation to this method. This could be owing to the nature of the arc selection algorithm navigating the separate overlap maps.

In all cases, dosimetric comparison relative to the standard arc template were calculated as the maximum absolute dose difference. One aspect that this comparison does not account for is the tolerance doses of the OARs used in this research. However, when comparing planning methodologies strictly in terms of maximum absolute dose differences, tolerance doses were not deemed essential to consider. For example, whether a four‐arc cranial template plan yielded OARs whose maximum doses were higher or lower than accepted tolerance doses, the comparison with a planning technique that was able to reduce those maximum doses would still be considered relevant for this research.

Evidenced by the example in Figure 3, and the summary in Table 1, the 4π optimized arc trajectories lead to plans that on average outperformed the standard cranial four‐arc template. However, no differences inside each class were significant according to a Wilcoxon signed rank test. Exceptions were found within the standard error for some OARs, indicated by positive values whose error bars did not cross below the zero line. These exceptions were also prevalent in classes 5 and 6 for trajectories generated using the anatomically informed template maps of Figure 2e,f, which occupied the posterior anatomical segment, including the occipital lobe, of the brain (Table 1 and Supplementary Material). Without many OARs to overlap in the BEV due to the larger distances between OARs and targets in these cases (demonstrated by the dark blue bands of Figure 2e,f), it is feasible that the standard arc template would outperform the class solution in both segments. However, the patient‐specific solutions in these classes all outperformed the standard arc template and the anatomically informed template trajectories (Table 1 and Supplementary Material). Conversely, as noted in Figure 1, there is more potential for BEV overlap when placing a synthetic target in classes 1, 2, 3, and 4 due to increased proximity of OARs and targets.

Classes 1 and 2 occupy the largest segmentations in the frontal region of the brain, where there is more potential for BEV overlap with the optic nerves, eyes, and lenses, and this is reflected by high‐intensity yellow bands in Figure 2a,b. Nevertheless, in both cases, trajectories were chosen that aimed to avoid these regions. Classes 3 and 4 occupy the medial regions of the brain where there is more potential for brainstem and optic chiasm overlap. Asymmetries exist between the overlap maps of Figure 2c,d due to differences in the amount of brainstem and chiasm volumes included in the MNI segmentation.

Various levels of maximum dose reduction were measured; however the anatomically informed template trajectories and patient‐specific trajectories of classes 3 and 4 yielded similar maximum dose reductions compared to the standard arc template (Table 1 and Supplementary Material). This implies that class solutions in the medial anatomical segments could be sufficient for cranial SRS/SRT planning if fixed‐couch optimization is performed. This comparison is further evaluated in Figure 5, where a case with many proximal OARs was compared next to a case with no proximal OARs (class 1 versus class 6). In this case, class 6 did not require any specialized techniques to lower OAR doses, but class 1 did for all proximal OAR in question.

The amount of maximum dose reduction is also important; taking note of the limits on the vertical axes of Figure 3, we see a range of approximately −3 Gy to +1 Gy. The closer to zero that these maximum dose reductions are (e.g., left lens in Figure 3), the less consequential it is to choose optimized fixed‐couch trajectories, irrespective of whether a patient‐specific optimization or class solution optimization is being performed. The doses in most cases are already low; however the management of cranial metastases is moving to a situation of retreatment and chronic management; thus any improvement in dose to OARs is advantageous. Whether it's clinically significant in one treatment is not important as the cumulative dose incurred by an OAR is more important.

A limitation of this methodology in comparison to HyperArc^5^ concerns the inability for the anatomically informed template to account for multiple targets. This was outside of the scope of this research and would require modification to the initial simulation. Instead of taking the maximum intensity from each overlap map to construct the anatomically informed overlap map, a method would be required to find the most conservative estimate for an overlap map comprised of multiple targets in the same anatomical region. The HyperArc technology^5^ does not consider cranial anatomy in its solution, instead it relies more heavily on the VMAT optimizer.

Another possible limitation in our methodology concerns the creation of the class solution overlap maps that use a single (summed) OAR structure that comprised all OARs. This facilitated the calculation for total overlap in a BEV by significantly reducing the computation time compared to an overlap calculation for each OAR separately. With this method, only 243 overlap maps needed to be calculated, while the individual overlap calculation comprised 243 × 8 = 1944 overlap maps, thus increasing computation time from on the order of hours to on the order of days. The number 243 arises from the maximum allowable number of spherical targets that were able to fit in the MNI brain given a diameter of 2 cm, each spaced by 2 cm, and constrained to be entirely contained within the outer contour. Despite this, the individual overlap method of MacDonald et al.^8^ was used to completely replan and analyze one test case to justify the single OAR methodology, and this yielded similar results to the same case using the single OAR structure methodology (results not shown). It was thus concluded that in general, optimal trajectories for a class solution would be found in similar low‐cost regions of an overlap map constructed from a single summed OAR structure or from an overlap map constructed from all the OARs measured separately. Thus, the overlap calculation methodology here differed from MacDonald et al,^8^ and no individual OAR weightings were applied. For the purposes of developing a set of anatomically informed template trajectories for cranial SRS/SRT, these weightings were not needed but could be applied in the future to modify the navigation procedures.

Another limitation was this research did not consider how to deal with edge cases in the MNI segmentation. These cases would be where a spherical target could lie across two or more anatomical segments. When performing the segmentation of the MNI brain after filling it with spherical targets, the overlap calculation would be performed only on the portion of the target inside the specified segmented anatomy. However, if the patient‐specific target did occupy multiple anatomical segments, it would be unclear what class solution to use. This limitation is readily addressed by performing a 4π optimization on the patient‐specific map for that case, potentially implying that there is clinical utility in having the option for both an anatomically informed class solution and a patient‐specific solution. Alternatively, an anatomically informed class solution could be chosen based on which segment the target occupied the most.

A final limitation of the research is that all comparisons to the standard cranial four‐arc template^6^ begin at a disadvantage in terms of how many control points are given to the Eclipse VMAT optimizer. The OFIXED trajectories were constrained to find at most the best four fixed‐couch arcs that spanned an overlap map from top to bottom only once (360 control points where each span 1° of gantry rotation), whereas the standard arc template is not limited in being able to resample gantry angles in 4π space. We are handicapping ourselves in the sense that we are not sampling the space as thoroughly as the template; therefore we expect improvements in conformity and gradient indices if we create other navigation approaches that account for better sampling. This is reflected in the statistically significant superiority of the metrics in favour of the standard arc template in Figure 4 and Table 3. Nonetheless, the purpose of this research was to compare fixed‐couch trajectory optimization in the context of a class solution methodology, which was VMAT optimized with 360 control points, to the standard arc template. Plans that can be optimized with less control points that maintain plan quality while reducing maximum dose will inherently be delivered more efficiently.

## 5 CONCLUSION

This research served to present and evaluate a novel methodology for creating anatomically informed class solution template trajectories for cranial SRS/SRT and comparing them with patient‐specific trajectories. The class solutions were created using the MNI average brain,^19^ with anatomically informed template trajectories calculated using a modified noncoplanar optimization framework.^18^ The arc selection techniques compared in this research vary in terms of their complexity. In approximately 70% of all cases, maximum doses to OAR were reduced relative to the standard‐arc template by only changing the arc geometry based on an optimization method. Improvements in maximum dose reduction in favour of patient‐specific were further realized when comparing the more complex patient‐specific optimizations to the anatomically informed class solutions. Furthermore, the most complex method for arc selection (OFIXEDi) showed the largest number of statistically significant differences when compared to the least complex method (VMATi). This could imply a tradeoff between the efficiency and familiarity of a class solution, and the potential for plan quality improvement offered by patient‐specific arc selection.

## AUTHOR CONTRIBUTIONS

*Study conception, treatment planning, data analysis, software development (data analysis), and manuscript preparation*: John David Lincoln. *Study conception, software development (4π algorithm), and critical review of the manuscript*: Robert Lee MacDonald. *Software development (4π algorithm)*: Brian Little and Alasdair Sym. *Critical review of the manuscript*: Alasdair Syme. *Study conception, software development, (4π algorithm), and critical review of the manuscript*: Christopher Grant Thomas.

## CONFLICT OF INTEREST

All authors acknowledge support from Brainlab AG and the Atlantic Canadian Opportunities Agency (ACOA). JL also acknowledges the Canadian Institute for Health Research (CIHR) ‐ GSD 167032.

## Supporting information

Supporting Information

Supporting Information
