# Supplementary material for: Comparison of anatomically informed class solution template trajectories with patient‐specific trajectories for stereotactic radiosurgery and radiotherapy
Source: J Appl Clin Med Phys. 2022 Sep 2;23(11):e13765. doi: 10.1002/acm2.13765 (PMC9680573; doi:10.1002/acm2.13765)
Supplement: Supplementary file 1 — Supporting Information [file ACM2-23-e13765-s002.pdf]

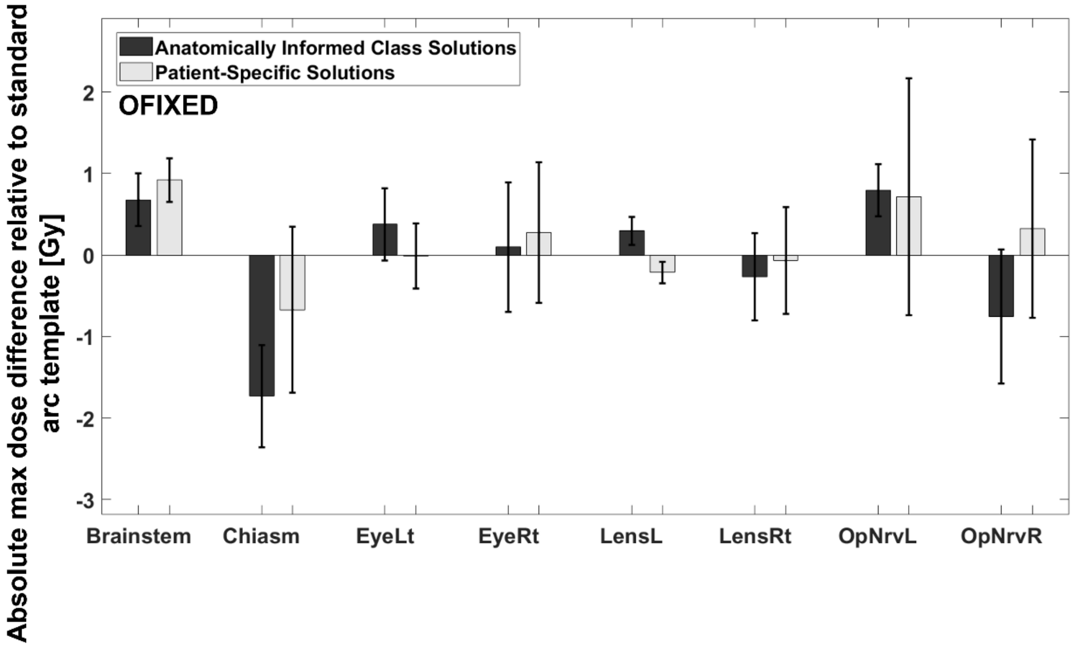

Supplemental Information 1: The results from anatomically informed class solution (Left Frontal segment). Dark bars indicate the results from the anatomically informed class solution trajectories, while the light bars indicate the results from patient specific trajectories. Dosimetric results are shown when applying trajectories using the OFIXED algorithm.

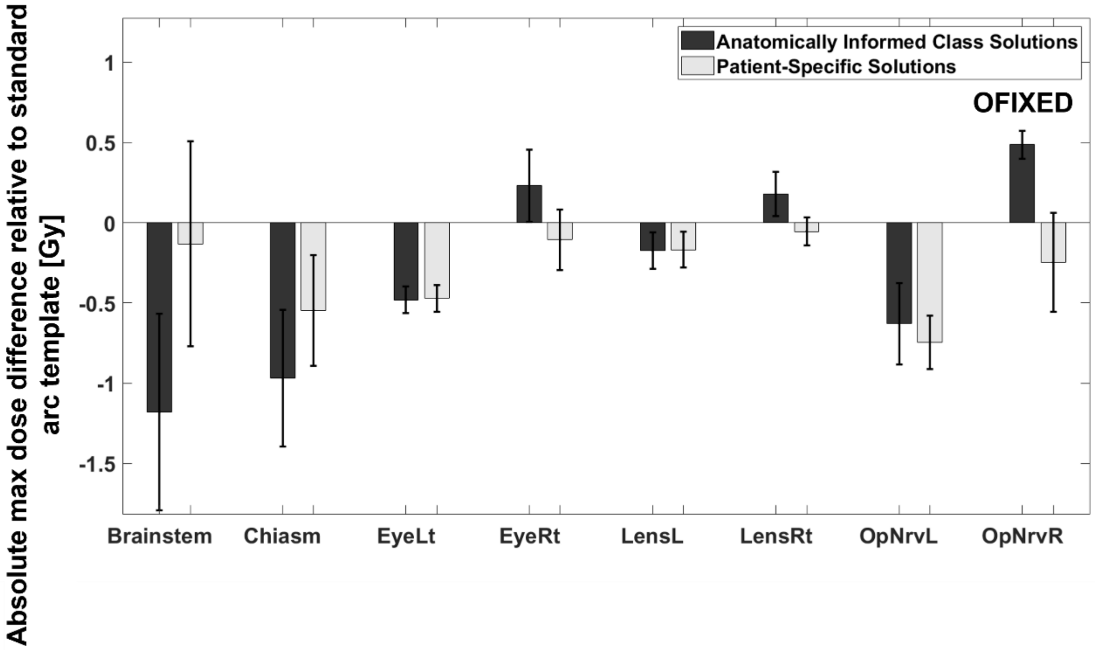

Supplemental Information 2: The results from anatomically informed class solution (Right Medial segment). Dark bars indicate the results from the anatomically informed class solution trajectories, while the light bars indicate the results from patient specific trajectories. Dosimetric results are shown when applying trajectories using the OFIXED algorithm.

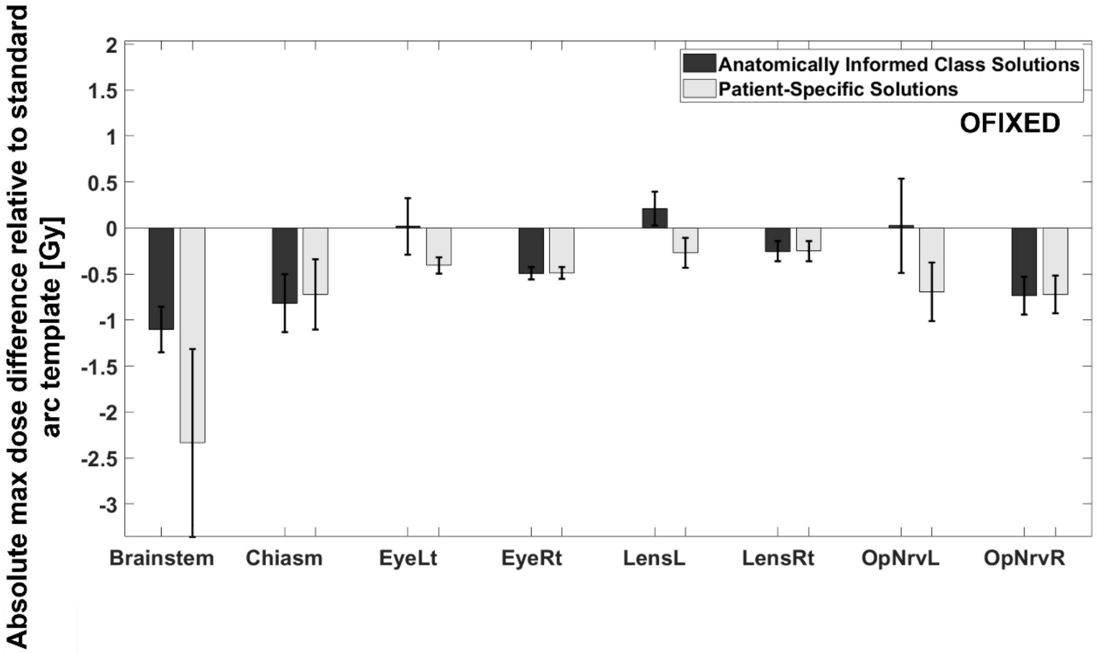

Supplemental Information 3: The results from anatomically informed class solution (Left Medial segment). Dark bars indicate the results from the anatomically informed class solution trajectories, while the light bars indicate the results from patient specific trajectories. Dosimetric results are shown when applying trajectories using the OFIXED algorithm.

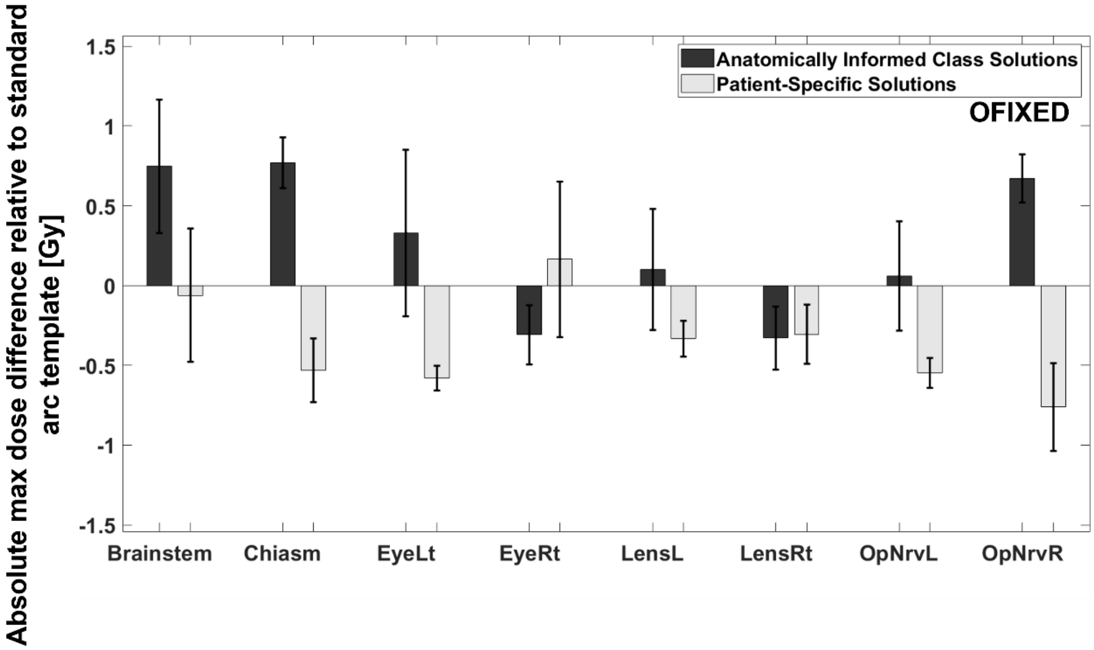

Supplemental Information 4: The results from anatomically informed class solution (Right Posterior segment). Dark bars indicate the results from the anatomically informed class solution trajectories, while the light bars indicate the results from patient specific trajectories. Dosimetric results are shown when applying trajectories using the OFIXED algorithm.

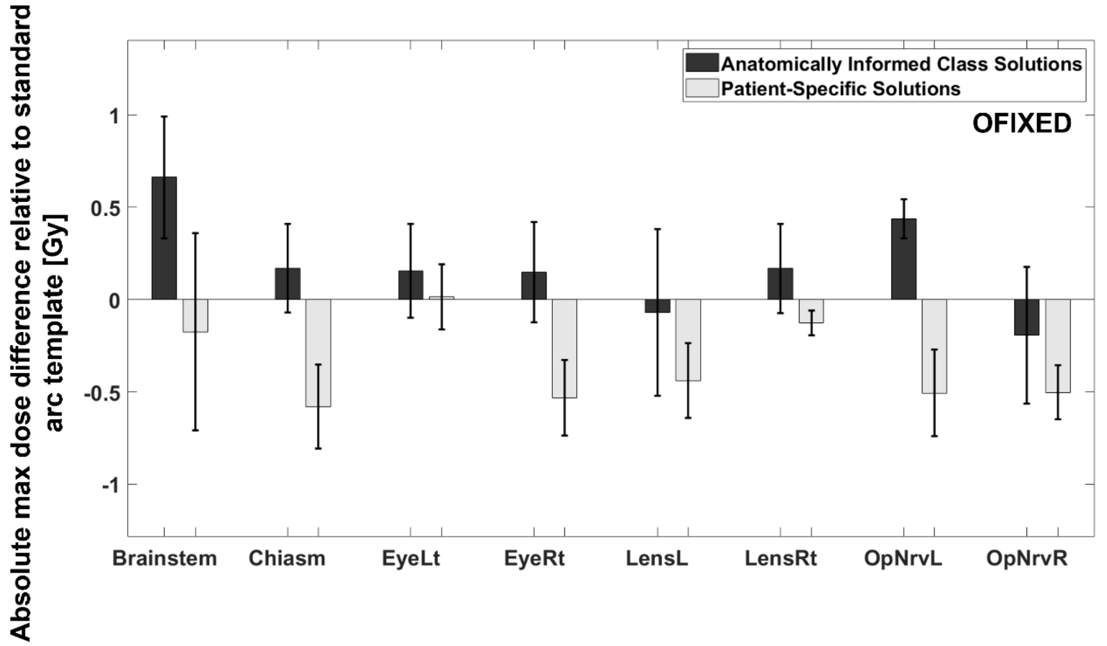

Supplemental Information 5: The results from anatomically informed class solution (Left Posterior segment). Dark bars indicate the results from the anatomically informed class solution trajectories, while the light bars indicate the results from patient specific trajectories. Dosimetric results are shown when applying trajectories using the OFIXED algorithm.
