# Supplementary material for: Comparison of anatomically informed class solution template trajectories with patient‐specific trajectories for stereotactic radiosurgery and radiotherapy
Source: J Appl Clin Med Phys. 2022 Sep 2;23(11):e13765. doi: 10.1002/acm2.13765 (PMC9680573; doi:10.1002/acm2.13765)
Supplement: Supplementary file 2 — Supporting Information [file ACM2-23-e13765-s001.pdf]

Supplemental Information 6: All plan technique comparisons for all metrics (max dose for all OARs) and plan quality metrics. Colour coding is as follows: All differences are calculated as (Technique 1 – Technique 2), where blue is positive and amber is negative. Significance according to the Bonferroni corrected Wilcoxon Signed Rank test accounting for multiple independent comparisons is shown in green for positive significance and red for negative significance.

Technique 1	Technique 2	Brainstem	Chiasm	Left Eye	Right Eye	Left Lens	Right Lens	Left Optic Nerve	Right Optic Nerve	V12 Gy	Monitor Units	Conformity Index	Gradient Index
**VMAT**	**OFIXEDc**	0.157	0.817	0.039	-0.107	-0.040	0.012	-0.017	-0.030	-0.262	-318.973	0.063	-1.088
**VMAT**	**OFIXEDi**	0.567	0.941	0.347	0.143	0.259	0.184	0.398	0.505	-0.596	-102.014	0.037	-0.679
**OFIXEDc**	**OFIXEDi**	0.410	0.123	0.308	0.251	0.299	0.172	0.415	0.535	-0.333	216.959	-0.026	0.409

Supplemental Information 7: All plan technique comparisons averaged for all metrics (max dose for all OARs) and plan quality metrics. Raw significance p-values calculated from a Wilcoxon Signed Rank on each of multiple independent comparisons.

Technique 1	Technique 2	Brainstem	Chiasm	Left Eye	Right Eye	Left Lens	Right Lens	Left Optic Nerve	Right Optic Nerve	V12 Gy	Monitor Units	Conformity Index	Gradient Index
**VMAT**	**OFIXEDc**	0.913	0.112	0.647	0.500	0.711	0.983	0.948	0.811	0.029	0.006	0.044	0.002
**VMAT**	**OFIXEDi**	0.306	0.002	0.005	0.372	0.000	0.012	0.003	0.005	0.001	0.420	0.112	0.016
**OFIXEDc**	**OFIXEDi**	0.102	0.396	0.215	0.170	0.022	0.071	0.022	0.016	0.006	0.102	0.744	0.679

Supplemental Information 8: OAR to target proximity information for each plan used in this research. Plan labels are given in the left most column (6 Classes each with 3 Targets). Proximity of each target to all OAR in question is given in centimeters.

Plan	Brainstem	Chiasm	Left Eye	Right Eye	Left Lens	Right Lens	Left Optic Nerve	Right Optic Nerve
**C1T1**	0.17	0.27	>1	>1	>1	>1	>1	0.58
**C1T2**	>1	>1	>1	>1	>1	>1	>1	>1
**C1T3**	>1	>1	>1	>1	>1	>1	>1	>1
**C2T1**	>1	0.38	>1	>1	>1	>1	0	>1
**C2T2**	>1	>1	>1	>1	>1	>1	>1	>1
**C2T3**	>1	0	>1	>1	>1	>1	0	0.83
**C3T1**	0	>1	>1	>1	>1	>1	>1	>1
**C3T2**	0	>1	>1	>1	>1	>1	>1	>1
**C3T3**	0.02	0.03	>1	>1	>1	>1	>1	>1
**C4T1**	0	>1	>1	>1	>1	>1	>1	>1
**C4T2**	>1	>1	>1	>1	>1	>1	>1	>1
**C4T3**	0.05	>1	>1	>1	>1	>1	>1	>1
**C5T1**	0.03	>1	>1	>1	>1	>1	>1	>1
**C5T2**	0.63	>1	>1	>1	>1	>1	>1	>1
**C5T3**	>1	>1	>1	>1	>1	>1	>1	>1
**C6T1**	0.3	>1	>1	>1	>1	>1	>1	>1
**C6T2**	>1	>1	>1	>1	>1	>1	>1	>1
**C6T3**	>1	>1	>1	>1	>1	>1	>1	>1

Supplemental Information 9: All plan technique comparisons for all metrics (max dose for all OAR) and plan quality metrics, filtered by OARs that had a proximity consideration given in supplemental information 8. The number of plans remaining with a proximity consideration are given in the numbers below the respective OAR columns (e.g - 9 plans have proximal brainstems according to Supplemental Information 8). Colour coding is as follows: All differences are calculated as (Technique 1 – Technique 2), where blue is positive and amber is negative. Significance according to the Bonferroni corrected Wilcoxon Signed Rank test accounting for multiple independent comparisons is shown in green for positive significance and red for negative significance.

Technique 1	Technique 2	Brainstem	Chiasm	Left Eye	Right Eye	Left Lens	Right Lens	Left Optic Nerve	Right Optic Nerve	V12 Gy	Monitor Units	Conformity Index	Gradient Index
VMAT	OFIXEDc	0.632	3.324	0.039	-0.107	-0.040	0.012	-0.696	0.240	-0.262	-318.973	0.063	-1.088
VMAT	OFIXEDi	1.059	2.279	0.347	0.143	0.259	0.184	-1.502	-0.248	-0.596	-102.014	0.037	-0.679
OFIXEDc	OFIXEDi	0.427	-1.046	0.308	0.251	0.299	0.172	-0.806	-0.488	-0.333	216.959	-0.026	0.409
		9*	4*					2*	2*				
